# Reaction-Dependent Structure–Activity Relationships in Penta-Twinned Palladium Nanocrystals

**DOI:** 10.3390/nano16191218

**Published:** 2026-09-27

**Authors:** Hojin Ahn

**Affiliations:** Department of Chemistry, Konkuk University, Seoul 05029, Republic of Korea; hojinahn@konkuk.ac.kr

**Keywords:** palladium nanocrystals, twinned nanostructures, electrocatalysis, oxygen reduction reaction, ethanol oxidation reaction, formic acid oxidation reaction, structure–activity relationships

## Abstract

Structural and electronic descriptors are widely used to rationalize electrocatalytic activity, but their predictive relevance can depend on the reaction pathway and adsorbed intermediates involved. Systematic comparisons of this reaction dependence using the same well-defined Pd nanostructures remain relatively limited. Here, two structurally related penta-twinned Pd nanocrystals, penta-twinned Pd nanosheets and decahedral nanocrystals, were comparatively evaluated for the oxygen reduction reaction, ethanol oxidation reaction, and formic acid oxidation reaction. For ORR, the more downshifted *d*-band center of penta-twinned Pd nanosheets accompanied higher intrinsic activity. In contrast, penta-twinned Pd nanosheets and decahedral nanocrystals exhibited nearly identical EOR-specific activities, together with closely comparable CO-stripping peak potentials. For FAOR, the activity order was reversed, with decahedral nanocrystals showing higher specific activity than penta-twinned Pd nanosheets. These contrasting trends demonstrate that the catalytic consequences of surface geometry and electronic structure depend on the adsorbates and elementary reaction pathways involved, highlighting the need to interpret catalyst descriptors in a reaction-specific context.

## 1. Introduction

The electrocatalytic properties of noble metal nanocrystals are governed not only by their elemental composition but also by the atomic arrangement and electronic structure of their surfaces [1,2,3]. At the nanoscale, control over particle shape provides a practical means of regulating the exposed crystallographic facets, the density of low-coordination sites, and the local strain environment, all of which influence the adsorption and transformation of reaction intermediates [1,4,5]. Palladium (Pd) is a particularly instructive platform for investigating such structure–activity relationships because its low-index surfaces exhibit markedly different adsorption characteristics and catalytic activities, while the face-centered cubic (fcc) lattice of Pd can accommodate a variety of multiply-twinned and anisotropic nanostructures [6,7,8,9]. Consequently, Pd nanocrystals with well-defined facets have been widely used as model catalysts for understanding how surface geometry and electronic structure govern electrocatalytic behavior.

Among these structural variables, lattice strain and twinning have attracted increasing attention because they can modify catalytic properties without altering the elemental composition of the surface [10,11,12,13]. Compressive or tensile strain changes the overlap of Pd *d* orbitals and shifts the *d*-band center relative to the Fermi level, thereby modifying the interaction between surface Pd atoms and adsorbed reaction intermediates [10,13,14,15]. Such strain effects have been exploited to tune oxygen-reduction activity in Pd-based catalysts, including alloyed and multiply-twinned systems [16,17,18,19]. In a previous work, freestanding Pd nanosheets containing five twin boundaries were developed as an unusual two-dimensional Pd architecture combining a fivefold-twinned framework with predominantly {110} surfaces [18]. Structural analyses revealed that the geometrical mismatch inherent to the penta-twinned configuration generates a characteristic strain distribution, while theoretical calculations indicated a downshift in the Pd *d*-band center relative to conventional Pd {110} surfaces. That study established penta-twinned Pd nanosheets as a useful model for examining twin-induced strain effects in Pd electrocatalysis; however, whether the structure–activity relationship established for ORR in this penta-twinned architecture is retained in reactions involving different adsorbates and elementary pathways remains unclear.

The oxygen reduction reaction (ORR), ethanol oxidation reaction (EOR), and formic acid oxidation reaction (FAOR) on Pd are representative structure-sensitive electrocatalytic reactions whose kinetics depend strongly on the adsorption, transformation, and desorption of surface-bound intermediates. Nevertheless, the identities of the relevant intermediates and the elementary reaction pathways differ substantially among these reactions. ORR proceeds through oxygenated intermediates such as OOH*, O*, and OH*, for which the strength of Pd–O interactions plays an important role [20,21,22,23]. EOR involves a more complex sequence of ethanol adsorption and dehydrogenation, the formation of ethoxy, acetyl, and other adsorbed carbonaceous species, and OH-assisted oxidation and removal of surface intermediates [24,25,26,27,28,29]. FAOR, in turn, involves formate- and carboxyl-related adsorbates whose formation, adsorption configuration, and subsequent transformation depend strongly on the local Pd surface environment [30,31,32,33,34,35]. Thus, although surface facet, lattice strain, and electronic structure can influence all three reactions, their relative contributions to catalytic activity may differ because each reaction involves distinct adsorbates and elementary reaction steps. Previous studies have demonstrated that the catalytic response of shape-controlled Pd nanocrystals can vary substantially between different electrochemical reactions and electrolyte environments. Nevertheless, cross-reaction comparisons remain relatively limited for structurally related Pd nanocatalysts in which twin-induced strain, electronic structure, and well-defined surface motifs coexist. Examining such systems across mechanistically distinct reactions can help clarify how the catalytic consequences of these structural characteristics change with the adsorbates and elementary reaction pathways involved.

Here, penta-twinned Pd nanosheets (Pd_PT_ NSs) and decahedral Pd nanocrystals (Pd_DH_ NCs) were employed as structurally well-defined penta-twinned Pd model catalysts. By evaluating the same catalyst pair across ORR, EOR, and FAOR and correlating their electrocatalytic activities with valence-band electronic structure and CO-stripping behavior, this study examines how the catalytic consequences of electronic structure and surface geometry evolve among distinct electrochemical reactions. This comparative approach provides a reaction-dependent view of structure–activity relationships in Pd electrocatalysis and highlights the limitations of transferring an activity descriptor established for one reaction directly to another.

## 2. Materials and Methods

### 2.1. Chemicals and Materials

Cetyltrimethylammonium chloride solution (CTAC, 25 wt% in water), cetyltrimethylammonium bromide (CTAB, ≥98%), potassium tetrachloropalladate(II) (K_2_PdCl_4_, 98%), sodium tetrachloropalladate(II) (Na_2_PdCl_4_, 98%), palladium(II) chloride (PdCl_2_, 99%), palladium(II) acetylacetonate (Pd(acac)_2_, 99%), citric acid (CA, ≥99.5%), sodium sulfate (Na_2_SO_4_, ≥99.0%, anhydrous), poly(vinylpyrrolidone) (PVP, average M_W_ = 55,000), poly(vinylpyrrolidone) (PVP, average M_W_ = 29,000), potassium hydroxide (KOH, 99.99%), diethylene glycol (DEG, 99%), pyridine (99%), acetonitrile (99.8%), diethyl ether (≥99.0%, anhydrous), benzyl alcohol (≥99.0%), acetone (≥99.5%), hydrochloric acid (HCl, 37%), formic acid (≥96%), ethanol (≥99.5%), Pd/C (10 wt% Pd), and Nafion perfluorinated resin solution (5 wt% in lower aliphatic alcohols and water, containing 15–20% water) were purchased from Sigma-Aldrich (St. Louis, MO, USA). L-Ascorbic acid (AA, ≥99.5%) was purchased from DaeJung Chemicals & Metals (Gyeonggi-do, Republic of Korea). 1-Bromodocosane was obtained from Tokyo Chemical Industry Co., Ltd. (Tokyo, Japan). Perchloric acid (HClO_4_, 70%) was obtained from GFS Chemicals (Delaware, OH, USA). All chemicals were used as received without further purification. Ultrapure water (resistivity > 18.2 MΩ cm) was used for the preparation of all aqueous solutions.

### 2.2. Synthesis of the Pd_PT_ NSs

Pd_PT_ NSs were synthesized according to a previously reported procedure with minor modifications [18]. CTAC (3.5 mL, 12.5 mM) and K_2_PdCl_4_ (1 mL, 5 mM) solutions were first equilibrated at 25 °C in a water bath and then transferred to a 10 mL vial. Subsequently, 0.5 mL of an aqueous solution containing AA (60 mM) and CA (40 mM) was added to the vial. The reaction mixture was maintained at 25 °C for 30 min without stirring. The product was collected by centrifugation at 600× *g* for 30 min, washed twice with water, and finally redispersed in water for subsequent use.

### 2.3. Synthesis of the Pd_DH_ NCs

Pd_DH_ NCs were synthesized according to a previously reported procedure with slight modifications [9]. In a 50 mL vial, Na_2_SO_4_ (320 mg) and PVP-55000 (640 mg) were dissolved in 16 mL of DEG. The resulting solution was stirred in an oil bath at 105 °C for 20 min. Subsequently, 8 mL of a Na_2_PdCl_4_ solution in DEG (15.5 mg mL^−1^) was injected into the reaction mixture. The reaction was allowed to proceed at 105 °C for 4 h under continuous stirring. After completion of the reaction, the product dispersion was mixed with acetone at a volume ratio of 5:8 and collected by centrifugation at 3600× *g* for 25 min. The collected product was washed twice with water and finally redispersed in water for further use.

### 2.4. Synthesis of the Palladium Nanosheets with {110} Terraces (Pd_{110}_ NSs)

Pd_{110}_ NSs were prepared based on a previous report with minor modifications [7]. A stock solution of 1-docosylpyridinium bromide (C22-pyBr) was prepared prior to the synthesis. For this purpose, 1-bromodocosane (3.9 g) and pyridine (1.2 g) were dissolved in 50 mL of acetonitrile in a round-bottom flask and heated under reflux at 95 °C for 20 h. After completion of the reaction, the obtained material was purified by washing with diethyl ether and subsequently dried using a rotary evaporator (EYELA, Tokyo, Japan). Separately, 10 mM H_2_PdCl_4_ solution was prepared by dissolving PdCl_2_ in an aqueous HCl solution (20 mM) at 60 °C for 1 h. To synthesize Pd{110} NSs, 1.6 mL of the H_2_PdCl_4_ solution (10 mM) was mixed with 5 mL of an aqueous C22-pyBr solution (50 mM) in a 20 mL vial. An aqueous AA solution (1 mL, 300 mM) was then added to the mixture. The vial was kept in a convection oven at 35 °C for 4 h. The resulting products were collected by centrifugation at 7000× *g* for 10 min, washed twice with water, and finally redispersed in water for subsequent experiments.

### 2.5. Synthesis of the Palladium Nanosheets with {111} Terraces (Pd_{111}_ NSs)

Pd{111} NSs were prepared based on a previous report with slight modifications [8]. Pd(acac)_2_ (50 mg), PVP-29000 (160 mg), CTAB (185 mg), and benzyl alcohol (10 mL) were added to a round-bottom flask and mixed thoroughly until a homogeneous solution was obtained. The reaction mixture was then heated at 70 °C for 3 h under a continuous CO flow of 0.5 mL s^−1^. After completion of the reaction, the product dispersion was mixed with acetone at a volume ratio of 2:8, followed by centrifugation at 14,000× *g* for 15 min. The collected product was washed once with acetone and once with water and finally redispersed in water for subsequent use.

### 2.6. Characterizations

Transmission electron microscopy (TEM) images were acquired using an FEI Tecnai F30 Super-Twin microscope (Hillsboro, OR, USA) operated at an accelerating voltage of 300 kV. X-ray photoelectron spectroscopy (XPS) measurements were performed using a Thermo VG Scientific Sigma Probe spectrometer (Thermo Fisher Scientific, Waltham, MA, USA) equipped with an Al Kα X-ray source (1486.7 eV). The binding energies were calibrated with respect to the C 1s peak at 284.8 eV. The Pd content of the samples was quantified by inductively coupled plasma optical emission spectroscopy (ICP-OES) using an Agilent 5110 ICP-OES instrument (Santa Clara, CA, USA).

### 2.7. Electrochemical Measurements

The electrode geometry and catalyst loading were selected according to the measurement requirements of each reaction. A rotating disk electrode was required for ORR to provide controlled hydrodynamic mass transport, whereas stationary glassy carbon electrodes were used for EOR and FAOR measurements. Within each reaction, identical electrode preparation and catalyst-loading conditions were applied to all catalysts to ensure internally consistent comparisons.

All electrochemical measurements were performed at room temperature using a conventional three-electrode system connected to a CHI 760D potentiostat (CH Instruments, Austin, TX, USA). For ORR measurements, a rotating disk electrode (RDE) equipped with a 5 mm-diameter glassy carbon disk was used as the working electrode. For EOR and FAOR measurements, a 3-mm diameter glassy carbon electrode was employed. An Ag/AgCl electrode (saturated KCl) and a Pt wire were used as the reference and counter electrodes, respectively. Unless otherwise stated, all measured potentials were converted to the reversible hydrogen electrode (RHE) scale according to

E_RHE_ = E_Ag/AgCl_ + E°_Ag/AgCl_ (0.197 V) + 0.059· pH,

where the potentials are given in volts at 25 °C. Prior to electrochemical measurements, all Pd catalysts were supported on Vulcan XC-72 carbon black with a Pd loading of approximately 25 wt%.

For ORR measurements, a catalyst ink containing Pd at a concentration of 0.0571 μg μL^−1^ and Nafion at 0.000714% was prepared. A 35 μL aliquot of the catalyst ink, corresponding to 2 μg of Pd, was deposited onto the 5-mm diameter glassy carbon RDE and dried under ambient conditions. The prepared working electrode was electrochemically conditioned by repeated cyclic voltammetry (CV) cycling in N_2_-saturated 0.1 M KOH at a scan rate of 50 mV s^−1^ until a stable voltammogram was obtained. ORR polarization curves were subsequently recorded by linear sweep voltammetry (LSV) in O_2_-saturated 0.1 M KOH at a scan rate of 20 mV s^−1^ and a rotation rate of 1600 rpm. The polarization curves were corrected for the background current and uncompensated solution resistance (*i*R). The kinetic current (*i_k_*) was determined using the Koutecky–Levich equation:

1/*i* = 1/*i_k_* + 1/*i_d_*,

where *i* is the measured current and *i_d_* is the diffusion-limited current.

For EOR measurements, a catalyst ink containing 0.1 μg μL^−1^ Pd and 0.0025 wt% Nafion was used. A 10 μL aliquot of the ink, corresponding to a Pd loading of 1 μg, was deposited onto a 3-mm diameter glassy carbon electrode and dried under ambient conditions. The electrode was electrochemically conditioned by repeated CV cycling in N_2_-saturated 0.1 M KOH at a scan rate of 50 mV s^−1^ until a stable voltammetric response was obtained. EOR activity was then evaluated by CV in N_2_-saturated 0.1 M KOH containing 0.5 M ethanol at a scan rate of 50 mV s^−1^. Chronoamperometric (CA) measurements were performed at −0.1 V versus Ag/AgCl to record the time-dependent current response under EOR conditions.

For FAOR measurements, a catalyst ink containing 0.1 μg μL^−1^ Pd and 0.0025 wt% Nafion was used. A 10 μL aliquot of the ink, corresponding to a Pd loading of 1 μg, was deposited onto a 3-mm diameter glassy carbon electrode and dried under ambient conditions. The electrode was electrochemically conditioned by repeated CV cycling in N_2_-saturated 0.1 M HClO_4_ at a scan rate of 50 mV s^−1^ until a stable voltammogram was obtained. FAOR activity was subsequently measured by CV in N_2_-saturated 0.1 M HClO_4_ containing 0.5 M formic acid at a scan rate of 50 mV s^−1^. CA measurements were performed at 0.2 V versus Ag/AgCl to record the time-dependent current response under FAOR conditions.

For CO-stripping measurements, the catalyst surface was saturated with adsorbed CO by bubbling CO gas through 0.1 M KOH for 20 min while holding the working electrode at 0.1 V versus RHE. Subsequently, high-purity N_2_ was bubbled through the electrolyte for 20 min to remove excess dissolved CO. CO-stripping voltammograms were subsequently recorded in 0.1 M KOH at a scan rate of 20 mV s^−1^.

The electrochemically active surface area (ECSA) of the catalysts was determined from the charge associated with Pd oxide reduction in the cathodic CV scan according to

ECSA_PdO_ = Q_PdO_/q_PdO_ m_Pd_,

where Q_PdO_ is the integrated Pd oxide reduction charge, q_PdO_ is the charge density associated with the reduction of a Pd oxide monolayer, and m_Pd_ is the Pd loading on the electrode. A conventional q_PdO_ value of 424 μC cm^−2^ was applied consistently to all Pd catalysts [36]. To independently evaluate the reliability of the PdO-derived ECSA values, additional replicate measurements were performed using PdO reduction in 0.1 M KOH and 0.1 M HClO_4_, together with CO-stripping measurements. The CO-stripping-derived ECSA was calculated according to

ECSA_CO_ = Q_CO_/q_CO_ m_Pd_,

where Q_CO_ is the integrated CO oxidation charge and q_CO_ is the specific charge density for oxidation of a saturated CO adlayer on Pd. A value of 420 μC cm^−2^ was used for q_CO_. The resulting ECSA values and experimental error ranges are summarized in Table S1. The reaction-specific ECSA values used for activity normalization were determined under the corresponding ORR, EOR, and FAOR measurement conditions, whereas the measurements summarized in Table S1 were used to evaluate the reproducibility and method dependence of the ECSA determination.

## 3. Results and Discussion

### 3.1. Penta-Twinned Pd Nanostructures

The main catalyst pair was selected to retain a common fivefold-twinned structural motif while varying overall morphology and dominant surface orientation. Pd_PT_ NSs exhibit a thin, two-dimensional sheet morphology extending from a characteristic fivefold-twinned central region (Figure 1a,b). HRTEM images and the corresponding FFT patterns are consistent with the previously established penta-twinned Pd nanosheet structure, in which the nanosheet surface is dominated by {110} terraces [18]. The combination of a two-dimensional morphology and multiple coherent twin boundaries produces a structurally distinct Pd surface environment in which surface geometry and twin-induced lattice distortion coexist. In contrast, Pd_DH_ NCs exhibit a compact three-dimensional decahedral morphology with the characteristic fivefold symmetry of multiply-twinned face-centered cubic (fcc) Pd and are predominantly enclosed by {111} facets (Figure 1c,d). Although both Pd_PT_ NSs and Pd_DH_ NCs possess a fivefold-twinned framework and identical elemental composition, they differ substantially in dimensionality, dominant surface orientation and the local structural environment associated with the twin boundaries. This catalyst pair therefore provides a useful model system for examining how the electrocatalytic role of a common penta-twinned motif is modulated by the surrounding surface structure across different electrochemical reactions. The non-penta-twinned Pd nanosheets with {110} terraces (Pd_{110}_ NSs) and Pd nanosheets with {111} terraces (Pd_{111}_ NSs) were additionally employed as facet-controlled reference catalysts specifically for the EOR and CO-stripping measurements, and their representative TEM images are shown in Figure S1. These reference nanostructures provide a complementary basis for interpreting the EOR behavior of Pd_PT_ NSs and Pd_DH_ NCs in the context of facet-dependent Pd electrocatalysis.

### 3.2. ORR Activities

The ORR was first examined to compare the electrocatalytic behavior of the two penta-twinned Pd nanostructures in alkaline electrolyte. Pd_PT_ NSs and Pd_DH_ NCs were deposited onto carbon supports before measurements of their electrocatalytic properties (Figure S2). Commercial Pd/C was also examined as a benchmark catalyst. CVs obtained in N_2_-saturated 0.1 M KOH solution showed distinct regions of Pd oxide reduction (Figure S3). The ECSA values of the catalysts were calculated from the charges associated with the Pd oxide reduction region in the CVs. The ECSA values of Pd_PT_ NSs, Pd_DH_ NCs, and Pd/C were calculated as 19.92, 16.17, and 45.78 m^2^ g^−1^, respectively. Pd_PT_ NSs with a thin, two-dimensional morphology exhibited a slightly higher ECSA value compared to the Pd_DH_ NCs. The reliability of the ECSA determination was further evaluated using replicate PdO-reduction measurements in alkaline and acidic electrolytes and independent CO-stripping measurements, with the corresponding values summarized in Table S1. The ORR polarization curves obtained in O_2_-saturated 0.1 M KOH solution at 1600 rpm showed a clear positive shift in the order Pd/C < Pd_DH_ NCs < Pd_PT_ NSs (Figure 2a). The corresponding half-wave potentials (*E*_1/2_) were 0.863, 0.886, and 0.908 V versus RHE for Pd/C, Pd_DH_ NCs, and Pd_PT_ NSs, respectively, confirming the superior ORR activity of Pd_PT_ NSs. This activity trend is maintained after normalization by the ECSA, with the specific activity following the order Pd_PT_ NSs (3.88 mA cm^−2^) > Pd_DH_ NCs (2.14 mA cm^−2^) > Pd/C (0.557 mA cm^−2^) (Figure 2b). Pd_PT_ NSs also exhibit the highest mass activity among the three catalysts. The Tafel plots derived from the kinetic current densities are shown in Figure S4. Pd_PT_ NSs, Pd_DH_ NCs, and Pd/C exhibited Tafel slopes of 42.9, 38.5, and 48.1 mV dec^−1^, respectively. The relatively similar Tafel slopes suggest that the three catalysts exhibit broadly comparable ORR kinetic behavior within the analyzed potential region. Notably, despite the slightly smaller Tafel slope of Pd_DH_ NCs, Pd_PT_ NSs showed the most positive *E*_1/2_ and the highest specific activity, indicating that the enhanced ORR performance of Pd_PT_ NSs is not attributable simply to differences in the Tafel slope. In the previous study, the enhanced ORR activity of Pd_PT_ NSs relative to conventional {110}-rich Pd nanostructures was associated with the strain induced by the penta-twinned framework [18]. The present comparison with Pd_DH_ NCs, which also possess a fivefold-twinned structure but are predominantly enclosed by {111} facets, provides an additional basis for examining the relationship between penta-twinning, electronic structure, and ORR activity.

Valence-band XPS spectra were obtained to probe the electronic structures of the catalysts. The *d*-band centers of Pd_PT_ NSs, Pd_DH_ NCs, and Pd/C were determined to be −2.96, −2.76, and −2.35 eV relative to the Fermi level, respectively (Figure 2c). Thus, the progressive downshift in the *d*-band center in the order Pd/C > Pd_DH_ NCs > Pd_PT_ NSs corresponds qualitatively to the increase in ORR-specific activity in the reverse order (Figure 2d). A downshifted Pd *d*-band center has generally been associated with weakened adsorption of oxygenated intermediates, which can facilitate ORR kinetics when oxygen-containing species bind too strongly to the Pd surface [17,18,20,21,22]. The observed correlation is therefore consistent with an electronic contribution to the enhanced ORR activity of Pd_PT_ NSs. However, because the three catalysts also differ in surface orientation, morphology, and local atomic structure, the present comparison does not establish the *d*-band center as the sole determinant of ORR activity. Rather, the results indicate that the more strongly downshifted electronic structure of Pd_PT_ NSs accompanies their higher intrinsic ORR activity and extends the strain-related structure–activity relationship identified in the previous work to a comparison between distinct penta-twinned Pd architectures.

### 3.3. EOR Activities

In contrast to the ORR behavior, a different activity relationship emerges for alkaline ethanol oxidation. The ECSA values determined from Pd oxide reduction regions in Figure 3a for Pd_PT_ NSs, Pd_DH_ NCs, Pd_{110}_ NSs, Pd_{111}_ NSs, and Pd/C were 25.94, 15.55, 21.40, 58.41, and 74.49 m^2^ g^−1^, respectively. CVs obtained in N_2_-saturated 0.1 M KOH containing 0.5 M ethanol and normalized to the Pd masses are shown in Figure 3b. Pd_PT_ NSs exhibited the highest EOR activity (1.76 A mg^−1^), followed by Pd_{111}_ NSs (1.13 A mg^−1^), Pd_DH_ NCs (1.05 A mg^−1^), Pd/C (0.697 A mg^−1^), and Pd_{110}_ NSs (0.50 A mg^−1^). Because mass activity reflects both the accessible surface area and the intrinsic activity of the exposed Pd surface, ECSA-normalized specific activity provides a more direct basis for comparing the intrinsic catalytic behavior of these nanostructures.

CVs in N_2_-saturated 0.1 M KOH containing 0.5 M ethanol normalized to the ECSAs are shown in Figure 3c. Pd_PT_ NSs and Pd_DH_ NCs exhibited closely comparable EOR-specific activities of 6.79 and 6.75 mA cm^−2^, respectively. These values were substantially higher than those of Pd_{110}_ NSs (2.34 mA cm^−2^), Pd_{111}_ NSs (1.94 mA cm^−2^), and Pd/C (0.935 mA cm^−2^). Thus, the clear difference between Pd_PT_ NSs and Pd_DH_ NCs observed for ORR becomes largely absent for EOR, with the two penta-twinned catalysts showing nearly identical intrinsic activities. The additional *d*-band center measurements further demonstrate that EOR activity does not exhibit a simple monotonic dependence on this electronic descriptor. Pd_PT_ NSs and Pd_DH_ NCs show nearly identical EOR-specific activities, despite their *d*-band centers differing by 0.20 eV. Moreover, Pd_{111}_ NSs exhibit a more downshifted *d*-band center (−2.53 eV) than Pd_{110}_ NSs (−2.46 eV) (Figure S5), whereas the EOR-specific activity is higher for Pd_{110}_ NSs than for Pd_{111}_ NSs. These results indicate that the *d*-band center position alone is insufficient to account for the EOR activity of the present Pd catalysts.

This deviation is reasonable considering the multistep surface chemistry of Pd-catalyzed EOR [26,37,38,39]. In addition to the average electronic structure represented by the *d*-band center, EOR can be influenced by the local atomic arrangement of the surface, defect and edge environments, adsorption configurations of ethanol-derived intermediates, and the formation and availability of OH-containing species required for their oxidative removal. Consequently, these structural and adsorbate-related contributions can modify the activity trend expected solely from the *d*-band center. Short-term chronoamperometric responses under EOR conditions are provided in Figure S6a for reference.

To further examine whether the similar intrinsic EOR activities of Pd_PT_ NSs and Pd_DH_ NCs are associated with comparable behavior toward the oxidative removal of strongly adsorbed surface species, CO-stripping measurements were performed in 0.1 M KOH solution (Figure 4). The main stripping peaks of Pd_PT_ NSs and Pd_DH_ NCs appeared at 0.827 and 0.825 V versus RHE, respectively, whereas Pd_{110}_ NSs and Pd_{111}_ NSs exhibited slightly more positive peak potentials of 0.832 and 0.837 V, respectively. Commercial Pd/C was additionally examined as a benchmark under the same conditions and exhibited a peak potential of 0.856 V versus RHE. The small potential difference between Pd_PT_ NSs and Pd_DH_ NCs indicates that the two penta-twinned catalysts exhibit closely comparable CO-stripping behavior under the present conditions.

The CO-stripping peak potential should not be interpreted as a direct measure of CO binding strength alone, because the oxidation of adsorbed CO depends simultaneously on the structure of the CO adlayer, the formation and adsorption of OH-containing species, and the local atomic arrangement of the Pd surface [40,41]. Accordingly, the stripping peak is used here as an empirical indicator of the potential required for the principal oxidative removal of adsorbed CO. Among the shape-controlled Pd catalysts, the trend toward lower CO-stripping peak potentials for Pd_PT_ NSs and Pd_DH_ NCs relative to Pd_{110}_ NSs and Pd_{111}_ NSs is broadly consistent with their higher EOR-specific activities. This correspondence suggests that the ability to remove strongly adsorbed surface species may contribute to the enhanced EOR activity of the penta-twinned catalysts.

### 3.4. FAOR Activities

FAOR exhibits a distinctly different structure–activity relationship from that observed for ORR. For ECSA determination, CVs were obtained in N_2_-saturated 0.1 M HClO_4_ solution (Figure 5a). The ECSA values calculated from Pd oxide reduction regions of CVs for Pd_PT_ NSs, Pd_DH_ NCs, and Pd/C were 21.61, 16.03, and 58.02 m^2^ g^−1^, respectively. Owing to its substantially larger accessible surface area per unit Pd mass, commercial Pd/C exhibited the highest mass activity of 1.69 A mg^−1^, whereas Pd_PT_ NSs and Pd_DH_ NCs showed comparable mass activities of 0.732 and 0.706 A mg^−1^, respectively (Figure 5b). After normalization by ECSA, however, Pd_DH_ NCs showed the highest specific activity (4.41 mA cm^−2^), followed by Pd_PT_ NSs (3.39 mA cm^−2^) and Pd/C (2.91 mA cm^−2^) (Figure 5c). Thus, both penta-twinned Pd nanostructures exhibit higher intrinsic FAOR activities than commercial Pd/C, while Pd_DH_ NCs outperform Pd_PT_ NSs. Short-term chronoamperometric responses under FAOR conditions are provided in Figure S6b for reference.

This activity order contrasts with that observed for ORR, indicating that the more downshifted *d*-band center of Pd_PT_ NSs does not translate directly into enhanced FAOR activity. This reversal can be understood in the context of the distinct adsorption and reaction requirements of FAOR. Previous single-crystal studies have established a facet-dependent FAOR activity of Pd in the order Pd(110) < Pd(111) < Pd(100) [31], indicating that terrace orientation can contribute to the structure sensitivity of FAOR. However, the higher FAOR activity of Pd_DH_ NCs relative to Pd_PT_ NSs cannot be attributed solely to their predominantly {111} and {110} surface orientations, respectively. The two nanostructures also differ in dimensionality, edge and corner densities, strain distributions, and the local atomic environments associated with their twin boundaries, all of which can influence the adsorption configurations and transformation of FAOR intermediates. Choi et al. demonstrated that multiply-twinned Pd decahedra and icosahedra exhibited higher FAOR-specific activities than their single-crystalline counterparts enclosed by the same {111} facets [34]. Their DFT analysis further suggested that the influence of such local site geometry on FAOR intermediates can exceed the effect of strain alone. These findings suggest that the enhanced activity of Pd_DH_ NCs in the present study may arise from a reaction-specific combination of the {111}-dominated terrace structure, edge and corner densities, and the local atomic environments associated with the penta-twinned architecture. Because Pd_PT_ NSs and Pd_DH_ NCs differ not only in dominant facet but also in dimensionality, strain distribution, edge structure, and the way twin boundaries intersect the surface, the individual contribution of each structural feature cannot be isolated from the present comparison. Nevertheless, the reversal of the activity order demonstrates that the electronic trend associated with ORR is not directly transferable to FAOR, for which different surface ensembles and intermediate pathways become relevant. This interpretation is also compatible with previous reports of non-monotonic relationships between Pd electronic structure and FAOR activity [42,43], further indicating that a progressively downshifted *d*-band center does not necessarily lead to improved FAOR kinetics.

### 3.5. Cross-Reaction Comparison of Structure–Activity Relationships

As summarized in Figure S7 and Table S2, the ORR, EOR, and FAOR results reveal that the catalytic consequences of the structural differences between Pd_PT_ NSs and Pd_DH_ NCs depend strongly on the reaction environment. In ORR, the activity trend follows the valence-band electronic structure, with the more downshifted *d*-band center of Pd_PT_ NSs accompanying higher intrinsic activity. For EOR, however, the *d*-band center does not provide a monotonic description of the activity across the catalyst series. Pd_PT_ NSs and Pd_DH_ NCs exhibit nearly identical specific activities despite their different *d*-band centers, while Pd_{111}_ NSs possess a more downshifted *d*-band center than Pd_{110}_ NSs but exhibit lower EOR-specific activity. Their similarly low CO-stripping peak potentials in alkaline electrolyte further suggest that the oxidative removal of strongly adsorbed species contributes to their favorable EOR behavior. For FAOR, the activity relationship is reversed, demonstrating that the electronic trend observed for ORR is not directly transferable to a reaction involving a different set of adsorbates and elementary steps.

These observations should not be interpreted as indicating that electronic structure is important only for ORR or that surface geometry is uniquely important for FAOR. All three reactions are structure-sensitive and are influenced by both the electronic and geometric properties of the Pd surface. Rather, the present results indicate that different reactions respond differently to the same combination of structural features because they involve distinct adsorption configurations, intermediates, and surface reaction pathways. Therefore, neither the *d*-band center position nor a probe reaction such as CO-stripping should be regarded as a universal descriptor of Pd electrocatalysis. Instead, their relevance must be considered in the context of the specific adsorbates and elementary processes governing each reaction.

## 4. Conclusions

Two structurally distinct penta-twinned Pd nanocatalysts, Pd_PT_ NSs and Pd_DH_ NCs, were employed as a model system to examine how structure–activity relationships vary across different electrochemical reactions. Although both catalysts share a common fivefold-twinned framework, they differ in surface orientation, morphology, strain state, and electronic structure, providing a useful comparative model system for cross-reaction comparison. The ORR activity trend was consistent with the *d*-band center positions, whereas this relationship was not preserved for EOR and was reversed for FAOR. These contrasting activity relationships demonstrate that the catalytic consequence of a given structural or electronic characteristic cannot be considered independently of the reaction in which it operates. Instead, electronic structure, surface atomic arrangement, and adsorbate-mediated surface chemistry act cooperatively, with their relative influence determined by the intermediates and elementary pathways involved. The findings therefore emphasize the importance of interpreting catalyst descriptors in a reaction-specific context and caution against directly transferring a structure–activity relationship established for one electrochemical reaction to another.

## Figures and Tables

**Figure 1:**
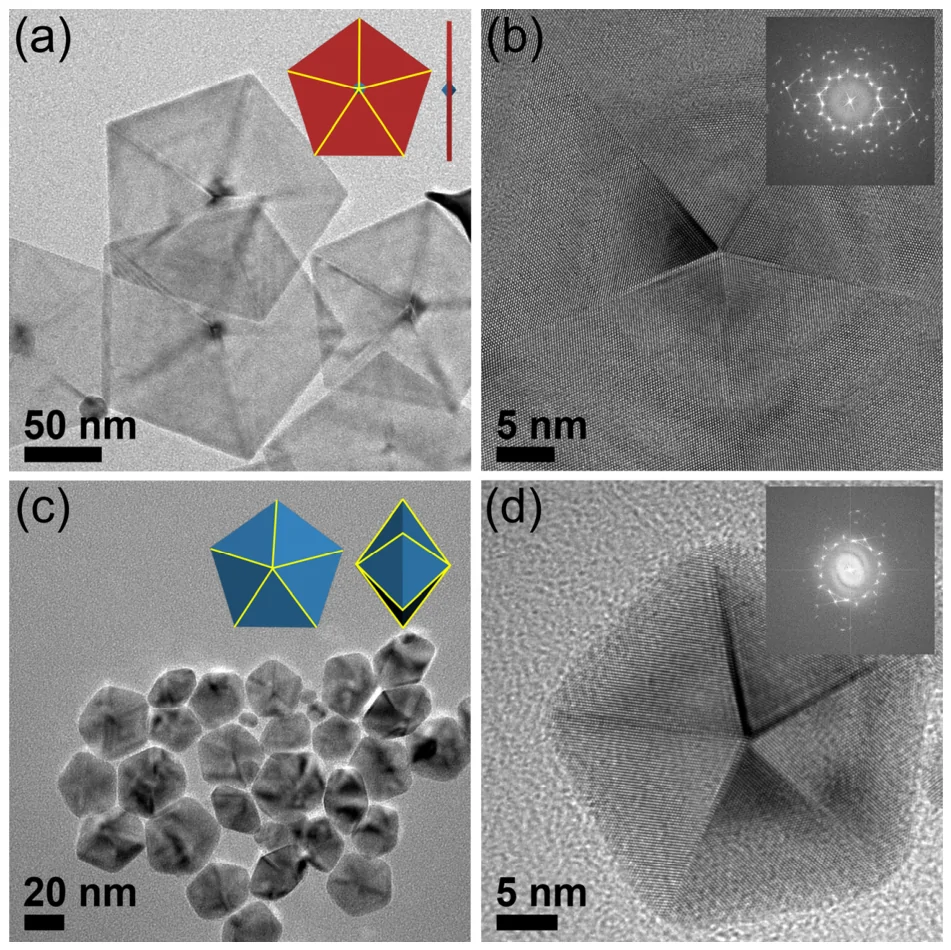
(**a**) TEM image of Pd_PT_ NSs. A schematic model of the Pd_PT_ NSs is shown in the inset. The red and blue colors indicate the {110} and {111} facets, respectively. The yellow line represents the twin boundary. (**b**) HRTEM image of Pd_PT_ NSs and the corresponding FFT pattern shown in the inset. (**c**) TEM image of Pd_DH_ NCs. A schematic model of the Pd_DH_ NCs is shown in the inset. The blue color indicates the {111} facets, and the yellow line represents the twin boundary. (**d**) HRTEM image of Pd_DH_ NCs and the corresponding FFT pattern shown in the inset.

**Figure 2:**
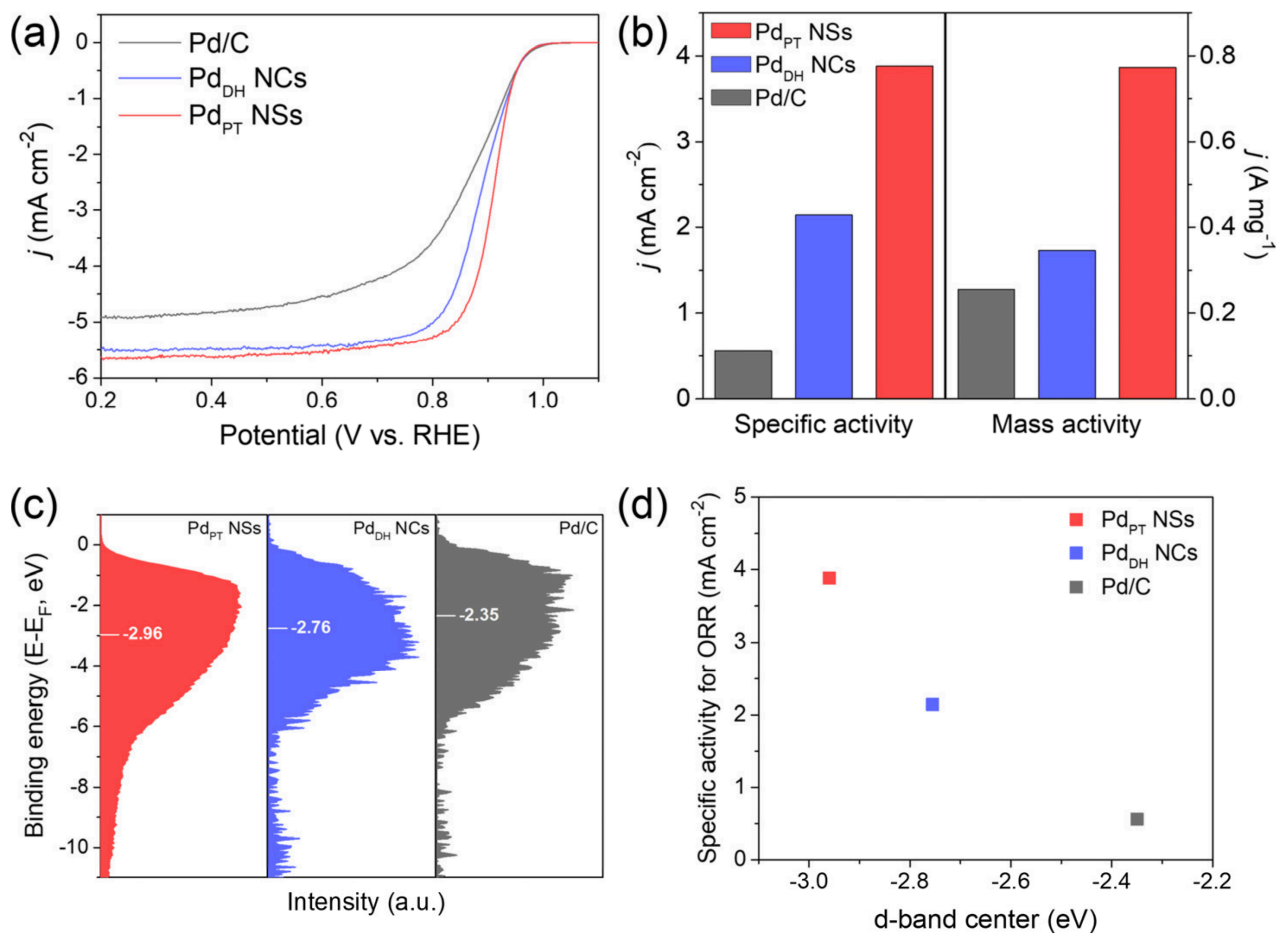
Electrocatalytic ORR performance of Pd_PT_ NSs, Pd_DH_ NCs, and Pd/C. (**a**) ORR polarization curves obtained in O_2_-saturated 0.1 M KOH solution at a scan rate of 20 mV s^−1^ and rotation rate of 1600 rpm. (**b**) Specific and mass activities of different catalysts at 0.9 V versus RHE. (**c**) XPS valence-band spectra of various catalysts. The position of the *d*-band center is indicated within each spectrum. E_F_ denotes the Fermi level. (**d**) Correlation plot between the *d*-band center and specific activity.

**Figure 3:**
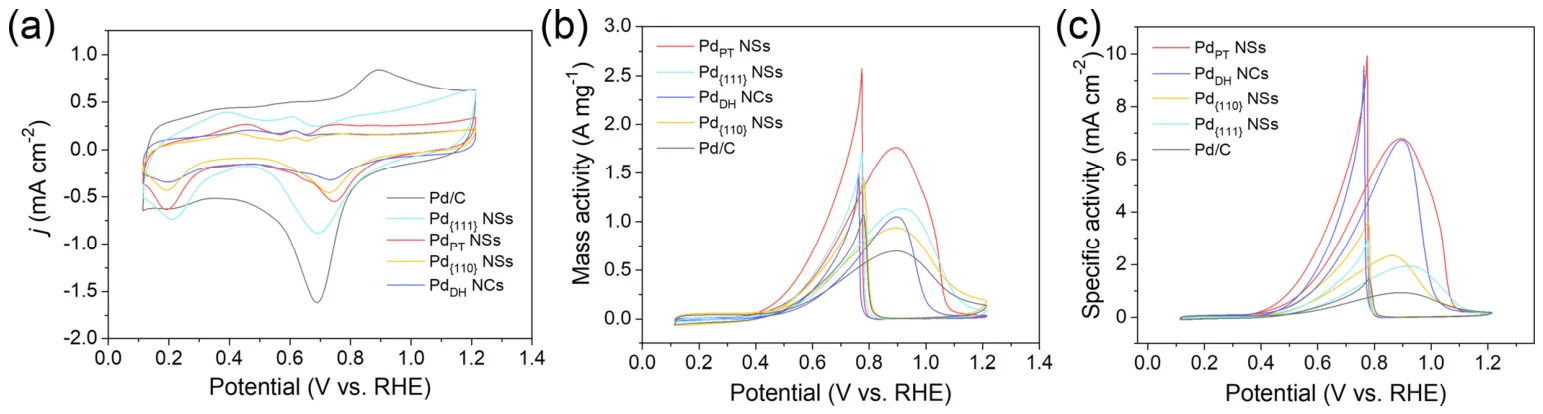
Electrocatalytic EOR performance of Pd_PT_ NSs, Pd_DH_ NCs, Pd_{110}_ NSs, Pd_{111}_ NSs, and Pd/C. (**a**) CVs of the catalysts in N_2_-saturated 0.1 M KOH at a scan rate of 50 mV s^−1^. (**b**) Pd mass-normalized CVs obtained in N_2_-saturated 0.1 M KOH + 0.5 M ethanol solution at a scan rate of 50 mV s^−1^. (**c**) ECSA-normalized CVs obtained in N_2_-saturated 0.1 M KOH + 0.5 M ethanol solution at a scan rate of 50 mV s^−1^.

**Figure 4:**
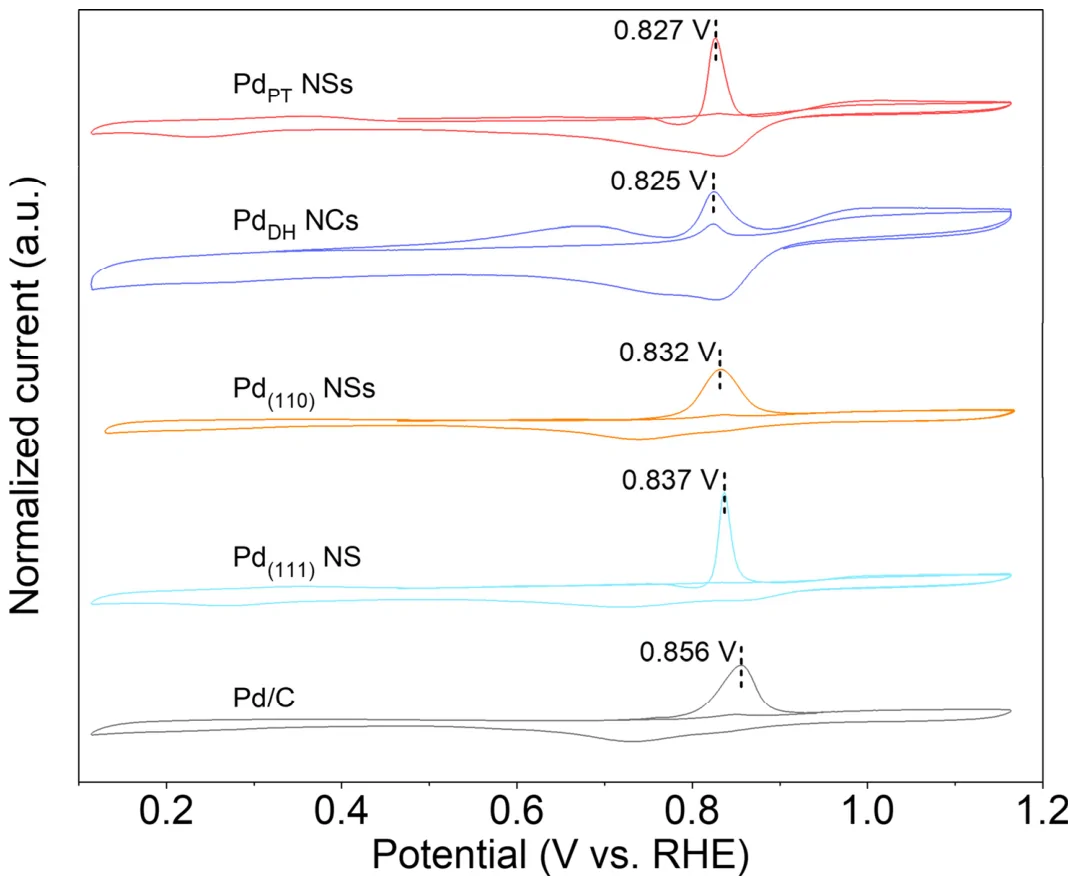
Normalized CO-stripping voltammograms of various catalysts in 0.1 M KOH at a scan rate of 20 mV s^−1^.

**Figure 5:**
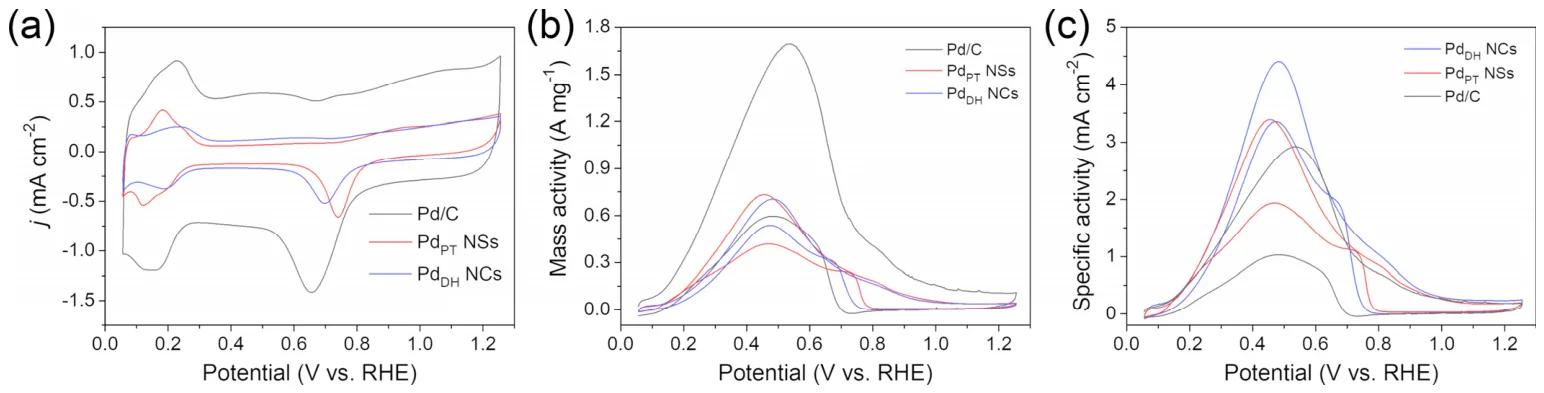
Electrocatalytic FAOR performance of Pd_PT_ NSs, Pd_DH_ NCs, and Pd/C. (**a**) CVs of the catalysts in N_2_-saturated 0.1 M HClO_4_ at a scan rate of 50 mV s^−1^. (**b**) Pd mass-normalized CVs obtained in N_2_-saturated 0.1 M HClO_4_ + 0.5 M HCOOH solution at a scan rate of 50 mV s^−1^. (**c**) ECSA-normalized CVs obtained in N_2_-saturated 0.1 M HClO_4_ + 0.5 M HCOOH solution at a scan rate of 50 mV s^−1^.

## Data Availability

The original contributions presented in this study are included in the article and in the Supplementary Information.

## Supplementary Materials

The following supporting information can be downloaded at https://www.mdpi.com/article/10.3390/nano16191218/s1: Figure S1 shows TEM images of Pd_{110}_ NSs and Pd_{111}_ NSs and corresponding FFT patterns. Figure S2 shows TEM images of the carbon-supported catalysts. Figure S3 shows CVs of the Pd_PT_ NSs, Pd_DH_ NCs, and Pd/C obtained in N_2_-saturated 0.1 M KOH for ORR. Figure S4 shows Tafel plots for ORR of the Pd_PT_ NSs, Pd_DH_ NCs, and Pd/C. Figure S5 shows XPS valence-band spectra of Pd_{111}_ NSs and Pd_{110}_ NSs, and their *d*-band centers. Figure S6 shows the short-term CA responses of the catalysts under EOR and FAOR conditions. Figure S7 shows a cross-reaction comparison of the relative specific activities of Pd_PT_ NSs and Pd_DH_ NCs for ORR, EOR, and FAOR. Table S1 compares the ECSA values determined from PdO reduction in 0.1 M KOH, PdO reduction in 0.1 M HClO_4_, and CO-stripping measurements, together with the corresponding experimental error ranges. Table S2 summarizes the ORR-, EOR-, and FAOR-specific activities, *d*-band centers, and CO-stripping peak potentials of the catalysts.
