# Supplementary material for: Reaction-Dependent Structure–Activity Relationships in Penta-Twinned Palladium Nanocrystals
Source: Nanomaterials (Basel). 2026 Sep 27;16(19):1218. doi: 10.3390/nano16191218 (PMC13667154; doi:10.3390/nano16191218)
Supplement: Supplementary file 1 [file nanomaterials-16-01218-s001.zip › nanomaterials-4577383-supplementary.pdf]

Supplementary Materials for

Reaction-Dependent Structure–Activity Relationships in Penta-Twinned Palladium Nanocrystals

Hojin Ahn

Department of Chemistry, Konkuk University, Seoul 05029, Republic of Korea; hojinahn@konkuk.ac.kr

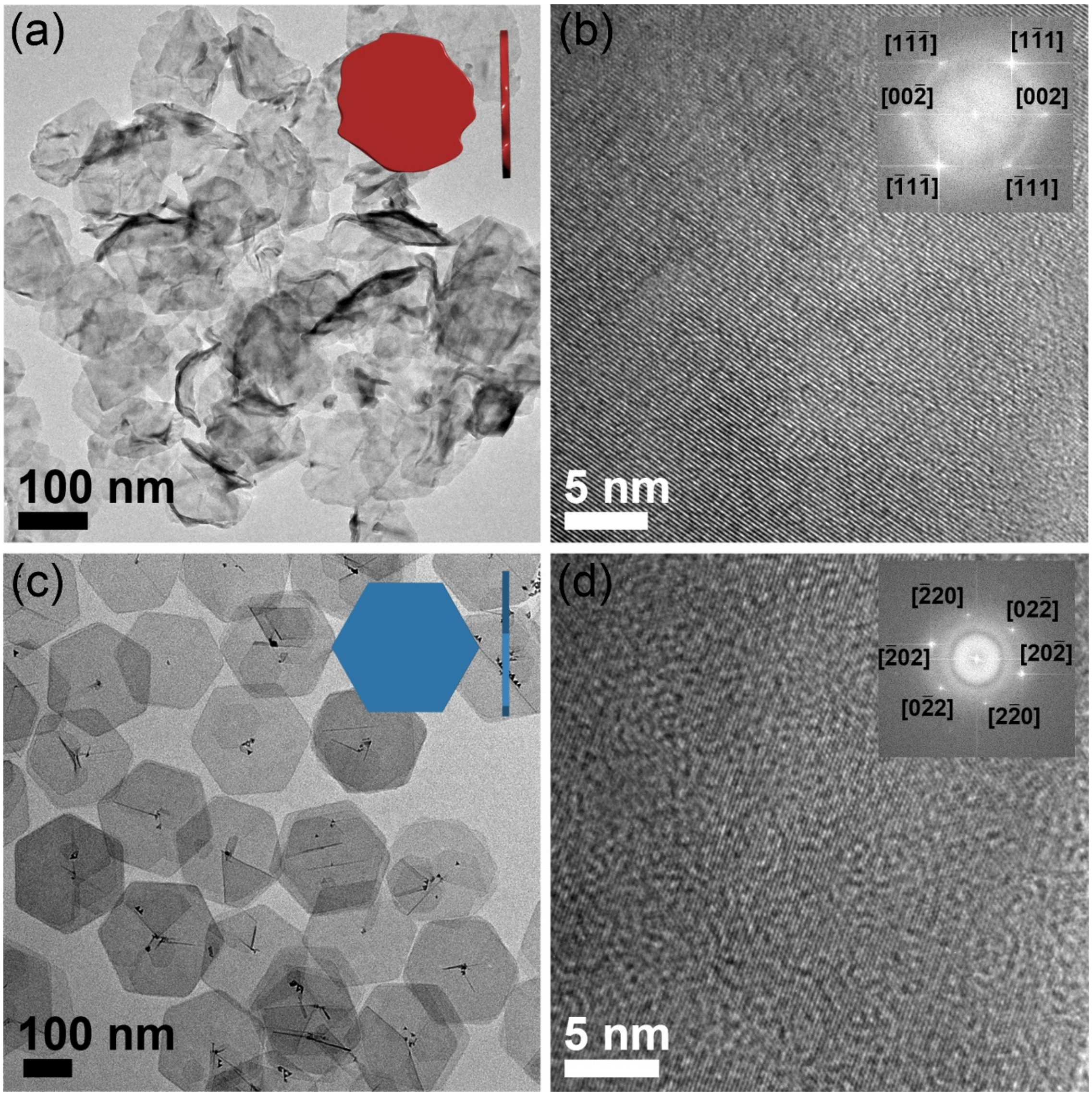

Figure S1. (a) TEM image of $Pd_{\{110\}}$ NSs. A schematic model of the $Pd_{\{110\}}$ NSs is shown in the inset; the red color indicates the {110} facets. (b) HRTEM image of $Pd_{\{110\}}$ NSs and the corresponding FFT pattern shown in the inset. (c) TEM image of $Pd_{\{111\}}$ NSs. A schematic model of the $Pd_{\{111\}}$ NSs is shown in the inset; the blue color indicates {111} facets. (d) HRTEM image of $Pd_{\{111\}}$ NSs and the corresponding FFT pattern shown in the inset.

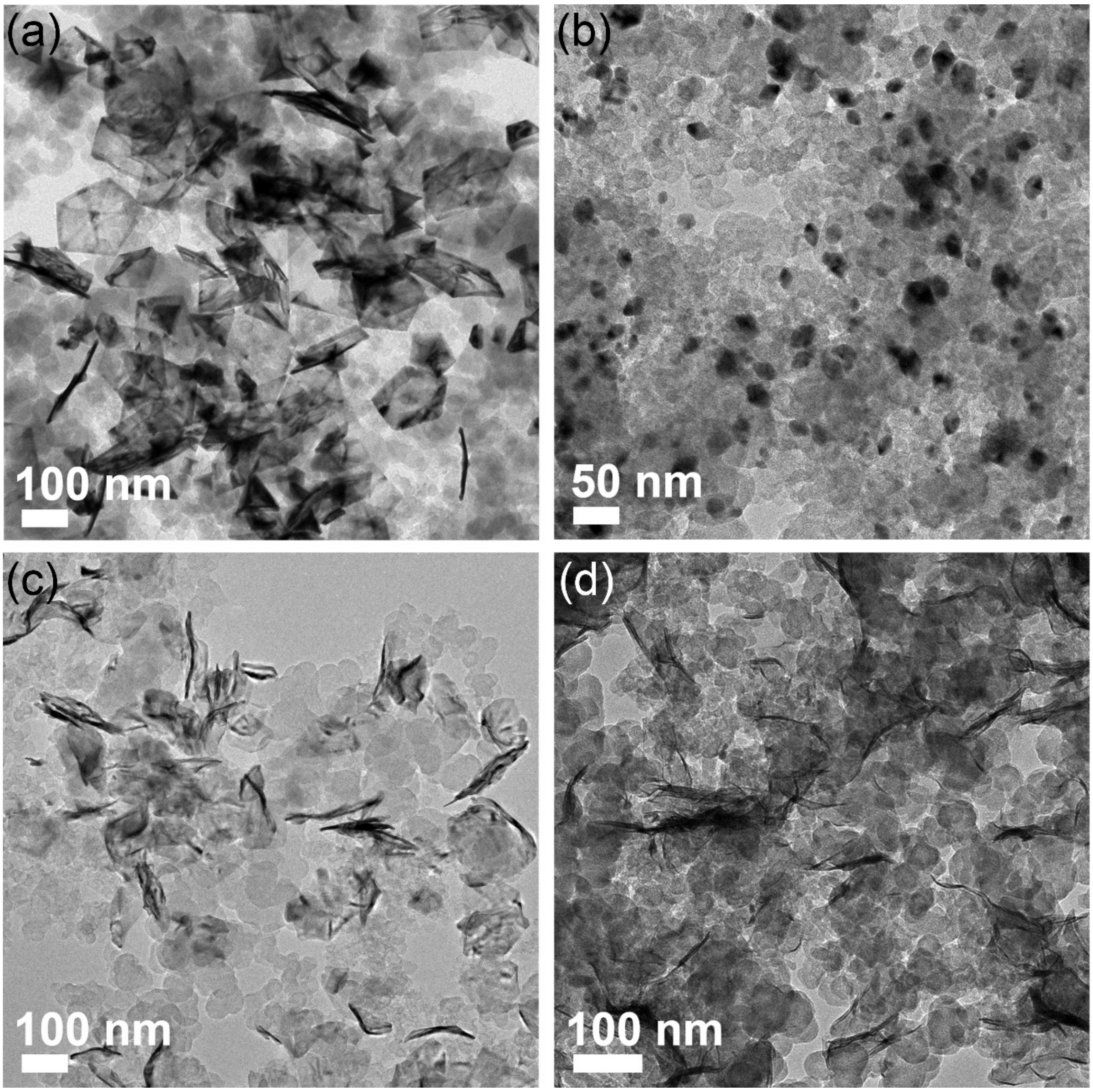

Figure S2. TEM images of (a) Pd_{PT} NSs, (b) Pd_{DH} NCs, (c) $Pd_{\{110\}}$ NSs, and (d) $Pd_{\{111\}}$ NSs after deposition onto Vulcan XC-72 carbon supports.

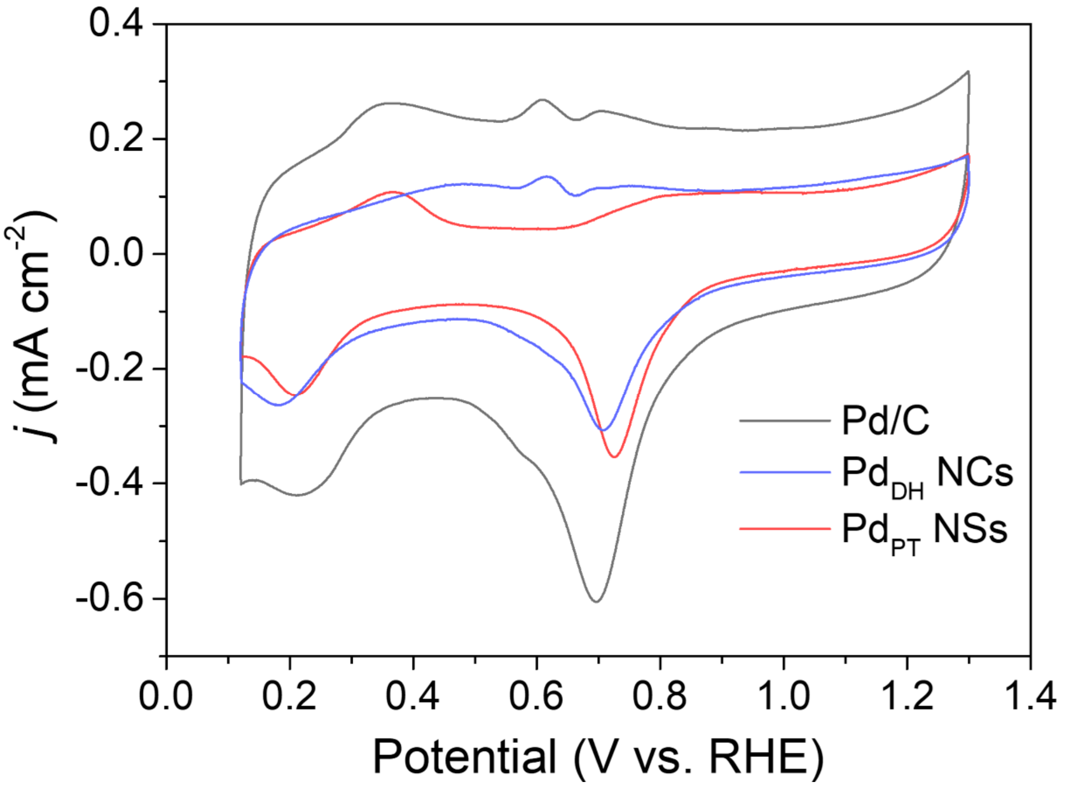

Figure S3. CVs of the catalysts in N_2-saturated 0.1 M KOH at a scan rate of 50 mV s^{-1}.

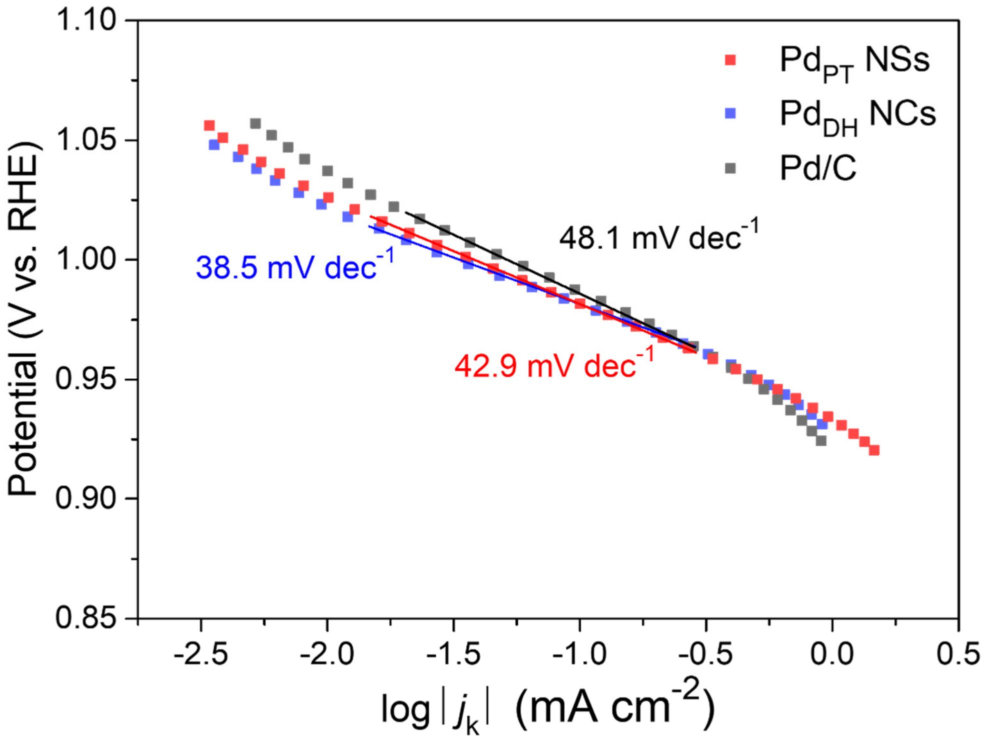

Figure S4. Tafel plots for ORR on Pd_{PT} NSs, Pd_{DH} NCs, and Pd/C derived from the kinetic current densities. The Tafel slopes obtained by linear fitting of the kinetic region were 42.9, 38.5, and 48.1 mV dec^{-1} for Pd_{PT} NSs, Pd_{DH} NCs, and Pd/C, respectively.

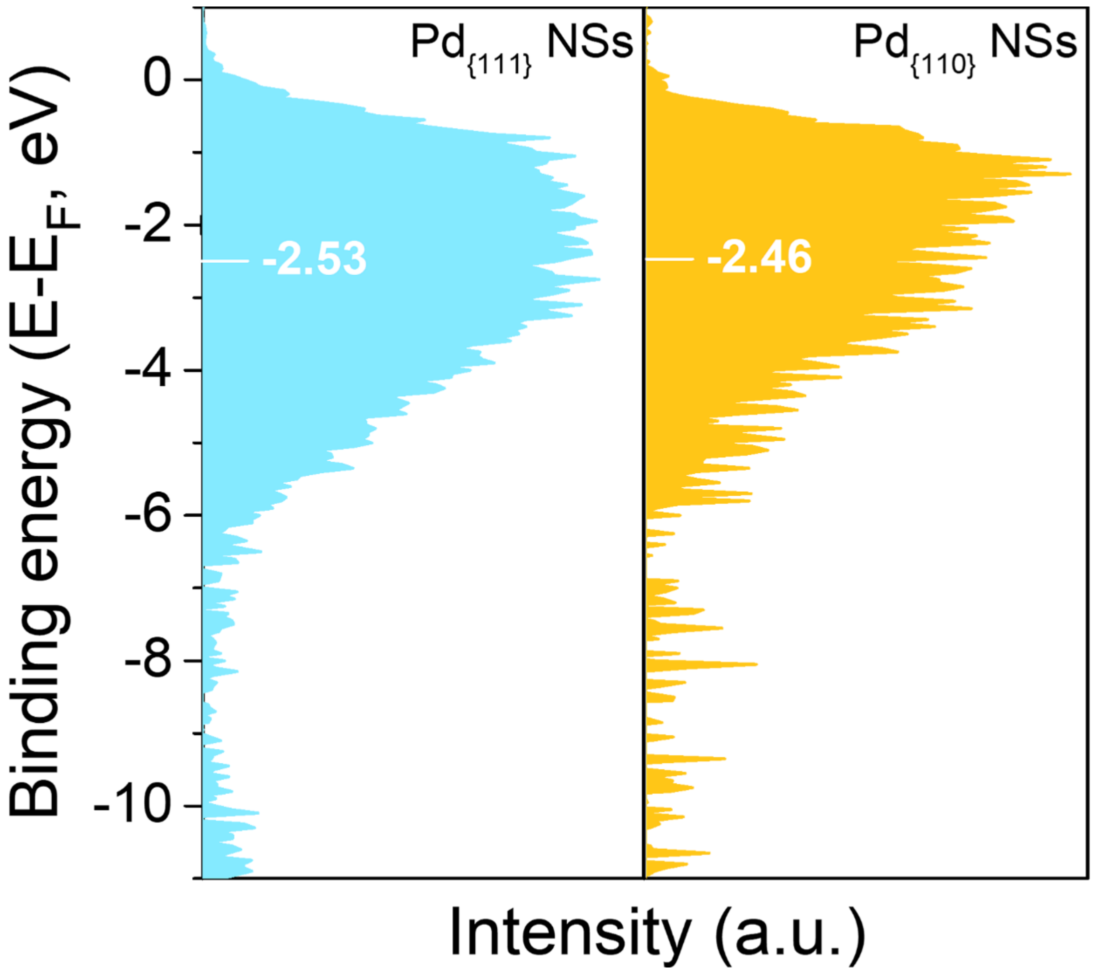

Figure S5. XPS valence-band spectra of $Pd_{\{111\}}$ NSs and $Pd_{\{110\}}$ NSs. The position of the d-band center is indicated within each spectrum. E_F denotes the Fermi level.

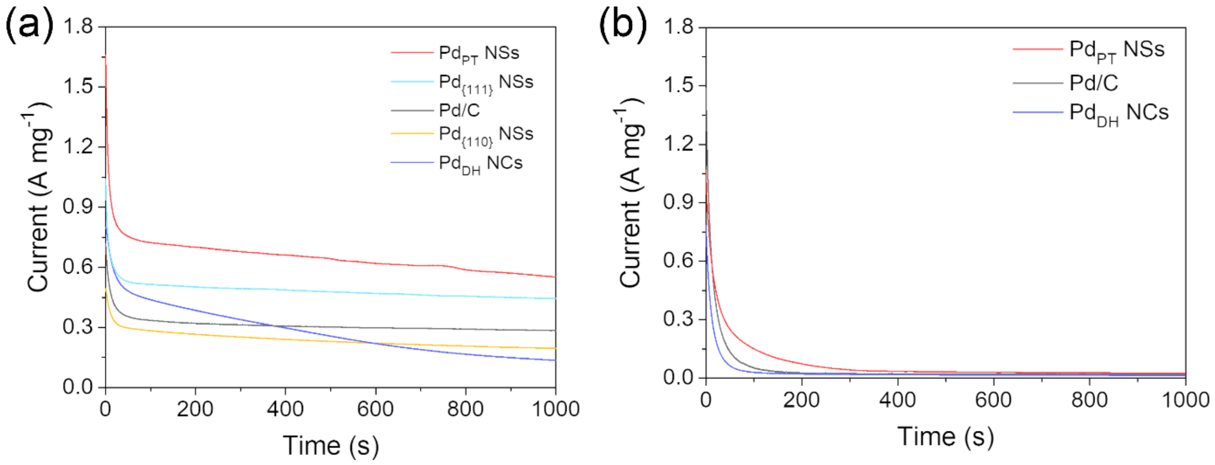

Figure S6. Pd mass normalized CA responses of the catalysts under (a) EOR conditions in N_2-saturated 0.1 M KOH containing 0.5 M ethanol and (b) FAOR conditions in N_2-saturated 0.1 M $HClO_4$ containing 0.5 M HCOOH.

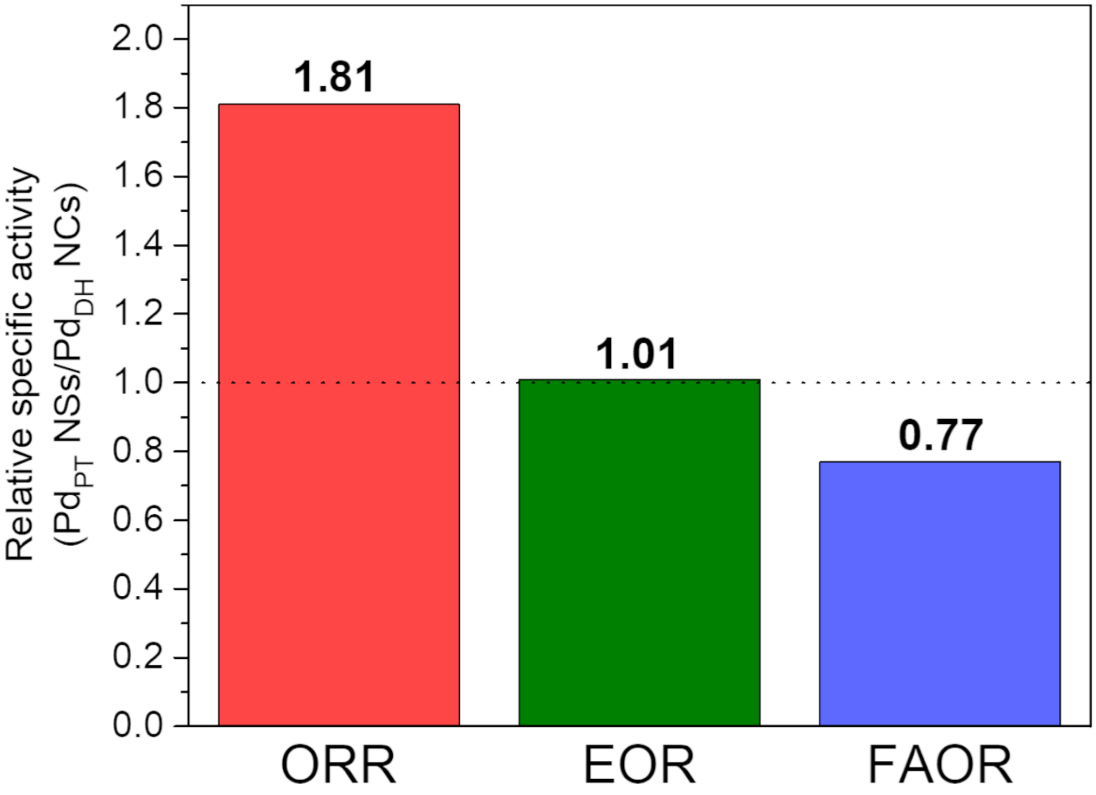

Figure S7. Cross-reaction comparison of the relative specific activities of Pd_{PT} NSs and Pd_{DH} NCs for ORR, EOR, and FAOR. The dashed line represents a relative activity ratio of 1.

Table S1. Comparison of the ECSAs determined from PdO reduction in 0.1 M KOH, PdO reduction in 0.1 M $HClO_4$, and CO-stripping measurements. Values are presented as mean ± standard deviation from three independently prepared electrodes.

Catalyst	ECSA from PdO reduction in 0.1 M KOH ($m^2\ g^{-1}$)	ECSA from PdO reduction in 0.1 M $HClO_4$ ($m^2\ g^{-1}$)	ECSA from CO-stripping ($m^2\ g^{-1}$)
Pd_{PT} NSs	23.97 ± 3.79	23.31 ± 4.86	23.17 ± 3.82
Pd_{DH} NCs	15.23 ± 0.40	15.73 ± 1.51	15.32 ± 1.22
$Pd_{\{110\}}$ NSs	20.17 ± 2.24	n.m.	21.52 ± 0.58
$Pd_{\{111\}}$ NSs	60.07 ± 4.45	n.m.	56.24 ± 6.27
Pd/C	58.84 ± 17.26	60.17 ± 14.91	69.53 ± 10.21

n.m., not measured.

Table S2. Summary of the ORR, EOR, and FAOR specific activities, *d*-band centers, and CO-stripping peak potentials of the Pd catalysts.

Catalyst	ORR specific activity (mA cm^{-2})	EOR specific activity (mA cm^{-2})	FAOR specific activity (mA cm^{-2})	*d*-band center (eV vs. E_F)	CO-stripping peak potential (V vs. RHE)
Pd_{PT} NSs	3.88	6.79	3.39	-2.96	0.827
Pd_{DH} NCs	2.14	6.75	4.41	-2.76	0.825
$Pd_{\{110\}}$ NSs	n.m.	2.34	n.m.	-2.46	0.832
$Pd_{\{111\}}$ NSs	n.m.	1.94	n.m.	-2.53	0.837
Pd/C	0.557	0.935	2.91	-2.35	0.856

n.m., not measured. $Pd_{\{110\}}$ NSs and $Pd_{\{111\}}$ NSs were employed as facet-controlled reference catalysts specifically for the EOR and CO-stripping analyses.
